# Popular Pastimes as Aids to Hospital Funds

**Published:** 1890-01-11

**Authors:** A. W. Webster

**Affiliations:** An Assistant Secretary to the London Hospital Saturday Fund


### Popular Pastimes as Aids to Hospital Funds.

By A. W. Webster, An Assistant Secretary to the London Hospital Saturday Fund.

### Football.

*(Continued from page 116)*

Football has formerly been more a Scotch than an English game, but the proverbial migratory habits of the Caledonians have enabled them to carry their favourite pastime into every corner of the country. The ardour with which they indulge in their kicking exercise has evidently become contagious, as we now find the game everywhere recognised as a popular winter sport. Indeed, in most of our large centres of population it is the game *par excellence* for eight months of the year.

Its advantage as an exercise is matter of dispute, many prominent physicians commending it as healthy and harmless, whilst others of high standing condemn it in unmeasured terms as the generator of heart disease and other deadly maladies. Both these statements appear to be extreme and probably refer to exceptions, but as I am totally unable to discuss the medical aspect of the subject, I merely accept the facts that innumerable football clubs exist and prosper, that players of high merit are found in every club of importance, and that few games are better supported by the public.

Experience has shown that the increasing keenness with which the game is played has been accompanied by a corresponding progressive proportion of casualties. The insurance companies have recently discovered this state of affairs, and have formulated systems of insurance specially applicable to the peculiarities of the game. What have the hospitals done in the matter? Their interest surely lies beyond setting the broken limbs, binding up the gaping wounds, and nursing and restoring to convalescence the numerous maimed players carried to their wards for treatment. Clearly, football players owe the hospitals a debt, which can and ought to be discharged by direct subscriptions from their club funds, and by indirect contributions derived from playing charity matches on the principle suggested under the head of cricket.

A good many clubs already play an occasional match in aid of a charity, but instead of arranging dates with first-class clubs and placing the fixtures on their usual cards issued at the beginning of the season, it is too often considered sufficient to play a "scratch" team, or any other middle-class club available on the shortest notice.

Generous subscribers in some localities have provided "charity shields" or "cups and medals" for annual competition, but as the rules of the game only allow the gate-money obtained at final and semi-final matches to be given to local charities (all proceeds of the earlier competitions being shared by opposing clubs), it will be seen that only a very few clubs make any sacrifice in the usual charity matches. This could be obviated by negotiating for a proportion of all gate-money drawn at matches in the earlier, as well as the later stages of the competition, but a plan likely to be more acceptable would be to try and get each club to make its own arrangements for charity fixtures, apart from the "prize" competitions referred to.

Not many years ago, the town of Sunderland knew comparatively little about football. A few enterprising enthusiasts got a fairly good "Association" team together, hired a field in a central position, and set themselves to work to popularise the game. In this they have been more than successful, for the chief club now only play with foemen worthy of their skill, which means the very best clubs in the kingdom. An offshot from the senior club also shows an almost equally bold front to all comers, having already defeated some of the "crack" clubs in England and Scotland. The attendance of spectators has gradually increased till it now varies from 3,000 to 12,000, according to the importance of the match and the meteorological conditions at the time, although the latter circumstance is treated with perfect indifference by many votaries of the "dribbling code." Much of the senior club's popularity was gained last season, when no less than four first-class charity matches were played. A single game between the senior club and its young rival, in January of this year, produced a gate of £168, the whole of which was divided between the local medical charities.

When suitable fields can be obtained, it is common to find officials of adjoining workshops, or particular classes of workmen, arrange friendly matches, and to hand any proceeds of the game to a worthy institution.

It matters little whether "Association" or "Rugby" be in the ascendant in any particular locality; the obligations and opportunities for supporting the hospitals are alike, and those interested should make it their duty to give the foregoing suggestions due attention.

### Cycling.

The popular nature of this pastime has already arrested the attention of our eagle-eyed legislators, and ere long they promise to send that bore of civilised communities—the tax collector—on the track of all lovers of cycling exercise. However this may be, the pastime is too well established to suffer more than a very temporary check, and we may look forward to a much-extended use of this handy and speedy mode of locomotion among all classes, and for mercantile and military purposes as well as those of exercise and pleasure.

The more ardent followers of this sport, as in others, will usually be found banded together in clubs, and my attention must first be devoted to these enthusiasts. I suppose most of my readers will have observed that clubs can hardly exist without races. Youthful aspirants seem always to demand opportunities of testing their speed and powers of endurance. Road races are generally resorted to where no enclosed track is available, but it will be obvious that no pecuniary results can be obtained without the means of taxing the sightseers. Some localities have been more enterprising and liberal than others in providing the necessary accommodation for developing "talent." All favoured spots possessed of railed-in cinder tracks can do much to aid local charities by organising tournaments annually, or as much more frequently as found convenient. I noticed in a newspaper report some months ago that considerably over £2,000 had been raised for local charities in Leicester within the past eight years by following this plan.

Sheffield last year initiated a similar movement, and notwithstanding the dull cold weather which prevailed on the Charity Cycling Tournament day, from 12,000 to 14,000 spectators patronised the event. Other examples might be cited, but the above seem sufficient to show the practicability of such schemes.

A few months ago a popular young bicyclist in the North of England was asked for a subscription to aid in the building of a local hospital. Feeling that he was unable to give any appreciable help from his private purse, he offered to attempt to break the five, ten, and twenty mile records on the local track, and give the entire proceeds of the gate to the object named. An energetic hospital secretary and workmen's committee soon organised the exhibition, securing the voluntary assistance of the best riders in the district as pace-makers, and the effort was crowned with monetary success.

The want of enclosed tracks will probably prevent the great majority of clubs from adopting the practices referred to, but I am sure many of them might overcome this difficulty by arranging with football associations to lay a cinder track round the outside of their playing boundary ropes, thus utilising the same enclosure for the double purpose of football and cycling. This is made all the more easy from the fact that the one is a winter and the other a summer pastime, and they would seldom interfere with each other's chief time for sport.

Need I say that hospitals deserve more than a passing remembrance or an occasional subscription from cyclists, if anything like adequate payment is to be made for the hundreds of disabled riders who are annually treated within the wards of such institutions? Indeed, it is doubtful if any pastime supplies more patients than this, and where cycling authorities fail to recognise their obligations, hospital officials may fairly endeavour to enlighten local wheelers as to their true position.

Let me conclude my notes on cycling by suggesting that our wheeling friends, whether banded together in clubs or not, might map out into sections and districts the outlying suburban residences and scattered villages, and carry out a systematic canvass on Hospital Saturday.

*(To be continued.)*

